# Between Syndemics and Structural Exploitation: Health Barriers of Migrant Agricultural Workers

**DOI:** 10.3390/ijerph22121783

**Published:** 2025-11-25

**Authors:** Yahel Kurlander, Nadav Davidovitch, Dani Filc, Zoe Gutzeit, Michal Tadjer, Einav Levy

**Affiliations:** 1 Faculty of Social Sciences and Humanities, Tel-Hai Academic College, Kiryat Shmona 1220800, Israel; yahelkur@telhai.ac.il; 2 Department of Health Policy and Management, Ben Gurion University, Beer Sheva 8410501, Israel; nadavd@bgu.ac.il; 3 Department of Politics and Government, Ben-Gurion University, Beersheba 8499000, Israel; dfilc@bgu.ac.il; 4 Physicians for Human Rights, Tel Aviv 6813509, Israel; zoe@phr.org.il; 5 Worker’s Hotline, Tel Aviv 6618608, Israel; michaltadjer@tauex.tau.ac.il; 6 Department of Social Work, Tel-Hai Academic College, Kiryat Shmona 1220800, Israel

**Keywords:** migrant labor workers, agricultural workers, health inequalities, syndemics, labor approach, occupational health, Israel, mental health

## Abstract

This study examines how Israel’s legal and institutional arrangements, including migration policies, health-insurance eligibility rules, and safety enforcement mechanisms, shape health risks for Thai migrant agricultural workers, and identifies policy levers to mitigate preventable harm. Using a legal policy analysis informed by syndemics and labor frameworks, we mapped laws, regulatory oversight, and enforcement chains, and synthesized evidence from worker surveys, affidavits, and civil society reports to link institutional structures with barriers experienced in workplaces and healthcare settings. The findings reveal that health inequalities stem from structural design rather than isolated failures, including exclusion from public insurance, dependence on employer-based plans, employer-tied visas, opaque injury-claim procedures, fragmented oversight, absence of occupational-health surveillance, limited language access, poor housing conditions, and weak inspections. These interlocking features reinforce one another, resulting in under-reporting, delayed medical attention, and cumulative physical and psychological harm. The study concludes that structural legal and administrative reforms—rather than individual adaptation—are required to address these inequities. Policy priorities include integrating migrant workers into public insurance or ensuring equivalent coverage, decoupling residency from single employers, guaranteeing multilingual access, establishing independent health monitoring, and enforcing safety, housing standards and other social determinants of health through transparent inspections, positive incentives and meaningful sanctions.

## 1. Introduction

In 2024, the International Labor Organization (ILO) estimated the global population of migrant workers at 167.7 million men and women [1]. A key distinction is made between high-skilled and low-skilled migrants. The latter are relegated to jobs that citizens avoid due to low pay and the “3D” nature of the work: dirty, dangerous, and demeaning [2]. Agriculture, the focus of this article, exemplifies this category. Other prominent examples include construction, shipyards, and factories [3]. Low-skilled migration usually takes place through Temporary Low-Skilled Migration Programs (TLMPs), which provide low wages and curtailed rights compared to local citizens [4]). In practice, TLMPs are often harmful, exposing migrant workers to exploitation, violations of human and labor rights, and even human trafficking [5,6,7,8,9].

### 1.1. Health Among Labor Migrants

The employment of low-skilled migrant workers is generally associated with a high incidence of work-related health problems [3,10]. These problems are attributed to long working hours, inadequate living conditions, and substandard sanitary facilities, including a lack of access to potable water [11]. Studies reveal a clear correlation between such conditions and both physical and mental health problems [5,12,13,14].

Poor mental health among migrant workers is linked to stressors such as separation from supportive communities, lack of medical response from employers, and workplace abuse. Studies consistently report high levels of psychological distress, depression, and anxiety among migrant workers, reflecting cumulative stressors of isolation, exploitation, and insecurity [12,15,16,17,18,19,20,21,22]. Beyond occupational hazards, the legal status of labor migrants strongly shapes their health. Undocumented workers face greater exploitation, poorer health outcomes, limited healthcare access, and heightened stress from raids and deportations [23,24,25]. Even legally employed agricultural migrants encounter barriers such as language, restricted system access, and lack of trust in healthcare institutions [26].

While previous research has extensively documented the prevalence of occupational injuries, poor living conditions, and psychological distress among migrant workers worldwide, less attention has been given to the structural and legal mechanisms that produce and sustain these adverse health effects. In particular, the intersection between institutional labor arrangements and syndemic interactions remains insufficiently theorized and empirically examined. This study addresses this gap by analyzing how Israel’s migration and health-insurance frameworks shape the cumulative health vulnerabilities of Thai agricultural workers through the combined lenses of the syndemics and labor frameworks. By integrating both approaches, we seek to incorporate the dynamics of temporal and spatial dimensions into our understanding of migrant workers’ physical and mental health, and help identify opportunities for change.

This study examines how Israel’s labor and health-insurance frameworks jointly shape the health and well-being of Thai agricultural workers. It aims to identify key health barriers, analyze the interaction between structural exploitation and syndemic vulnerabilities, and highlight policy implications for improving migrant workers’ protection and access to care.

### 1.2. Theoretical Framework

To examine this phenomenon thoroughly, we propose combining two different perspectives: the syndemics model and the labor approach to the paradigm of human trafficking.

The syndemics approach: The term syndemic describes the dynamic interaction of two or more diseases with social and environmental conditions that intensify their effects [27]. A syndemic involves: (1) co-occurring or sequential health conditions; (2) harmful interactions between them; and (3) socioeconomic, cultural, and environmental factors that exacerbate vulnerability [28,29]. Three models of syndemic interaction are identified: mutual causality, synergism, where combined effects exceed individual ones, and serial causality, which emphasizes cumulative risks through allostatic load [30]. These models demonstrate how clustered diseases mirror social inequalities and structural marginalization [27,29,31,32,33].

The labor approach to human trafficking: This approach offers an alternative to the human rights–criminal law model, which treats trafficking mainly as a criminal violation affecting limited cases [34]. In contrast, the labor approach contends that power imbalances between workers, employees, and the state create a labor market structure that perpetuates and exacerbates workers’ vulnerability to exploitation [35]. According to this approach, worker vulnerability stems from structural labor market inequalities in bargaining positions [34]. The labor approach thus shifts attention from individual harm to structural power disparities, highlighting economic coercion, commodification, restrictive immigration policies, and deregulated labor markets as key drivers of migrant workers’ vulnerability and exploitation. Moreover, the labor approach illuminates the role of migrant workers’ agency and power from below to contest exploitation [34,36,37,38,39,40,41].

Both the syndemics and labor approaches have been used to study labor migration 1 [7,29,42,43,44,45]. This study’s novelty lies in its integration of these two frameworks that are rarely examined together. Combining these perspectives makes it possible to link workers’ lived health experiences to the broader political–economic structures that shape them, clarifying how structural exploitation and interacting health burdens reinforce one another in Israel’s agricultural sector. While the syndemics model has been widely applied to explain how co-occurring diseases interact under conditions of social inequality, and the labor approach has focused on the institutional and economic mechanisms of worker exploitation, the combination of these frameworks allows for a more holistic analysis that bridges health and labor regimes. By conceptualizing migrant workers’ health not only as a product of biological and social interactions but also as an outcome of labor market structures and policy design, this study advances a multi-level perspective linking epidemiological patterns to legal institutional determinants. To our knowledge, this is the first empirical and conceptual application of this combined lens to migrant agricultural workers in Israel, and one of only a few studies in the world to use it as a joint explanatory framework for health inequities in temporary labor migration systems. Their combination may provide fresh insights, especially in the context of temporal and dynamic aspects of migration [46,47].

### 1.3. The Case of Agricultural Migrant Labor Workers (MLWs) from Thailand in Israel

Labor migration to Israel began after the 1967 Six-Day War, when Palestinians from the West Bank and Gaza increasingly entered the Israeli labor market to perform manual and semi-skilled jobs [48]. Until the late 1980s, construction and agriculture in Israel relied mainly on Palestinian labor. The first Intifada, subsequent security closures, the Oslo process, and neoliberal policies shifted the labor regime toward migrant workers from abroad, especially from Thailand, though Palestinians continued to participate in varying proportions. Overseas migrants entered agriculture and construction, where Palestinians had dominated, and eldercare, where they replaced Israeli workers in the late 1980s [49]. These remain the three main sectors of Israel’s labor migration programs today.

As of July 2025, Israel hosted more than 166,000 documented and 34,000 undocumented migrant workers. Of the documented, 61,000 worked in eldercare, 54,500 in construction, and 36,500 in agriculture, with smaller numbers in restaurants, hotels, industry, and services. 

Since the late 1980s, Israel’s agricultural sector has relied mainly on Thai workers, who for decades paid exorbitant recruitment fees amounting to debt bondage and trafficking. A 2012 bilateral agreement with Thailand established a new recruitment system that eliminated these fees [6,50]. The 7 October war disrupted bilateral agreements, leading to short-term private recruitment. Although the agreement has since been reinstated, studies are now investigating whether recruitment fees persist despite the ban [51].

Like other migrant workers in Israel, the MLWs from Thailand are excluded from the public health system because they are not considered residents of the state. Therefore, they are not included in the framework of the National Health Insurance Law-1994 and are unentitled to the health services the legislation provides. Under the Foreign Workers Law enacted in 1991 and the Foreign Workers Ordinance signed by the health minister in 2001, responsibility for the health of foreign workers was transferred to the private market: to employers, who are obligated to arrange health insurance for their migrant workers; and to private insurance companies, who sell employers policies tailored for foreign workers. These policies include a limited healthcare basket, primarily supplied by the same health maintenance organizations (HMOs) that provide services under the National Health Insurance Law. The HMOs provide services based on guidelines stipulated by the Foreign Workers Ordinance. Since 2014, only three insurance companies have sold insurance policies for foreign workers in Israel. Raijman & Kushnirovich (2019) [50] found that about a third of the workers were unaware of the fact that they had health insurance. Of those who knew they had insurance coverage, only 70% could say which HMO they belonged to.

When migrant workers first entered Israel, responsibility for their health was shifted to the private market, regulated by the Ministry of Finance [52]. Employers’ private insurance thus became the only source of routine care [53]. The Israeli Patient’s Rights Law guarantees emergency treatment for all persons regardless of status, but this applies only to urgent cases and does not replace access to regular community healthcare. Structural limitations, low awareness, and lack of state action undermine MLWs’ well-being and contribute to poor health. A study in Israel [54] found that 59% of Thai laborers working with pesticides were unaware of the risks, and 64.6% could not read safety labels. Such exposure causes acute symptoms (nausea, dizziness, headaches) and increases the risk of chronic respiratory, neurological, reproductive, and skin diseases, as well as cancer.

The Israeli agricultural sector offers a particularly revealing case through the experience of Thai MLWs, who constitute the largest group of foreign farmworkers in the country. Their situation reflects the convergence of multiple risk factors: hazardous occupational conditions, exclusion from the public health system, dependence on private insurance controlled by employers, and the broader vulnerabilities of temporary migration frameworks. Within this setting, the syndemic perspective elucidates how overlapping health burdens—physical injury, chronic illness, and psychological distress—are compounded by adverse living and working conditions. At the same time, the labor approach underscores how these vulnerabilities are entrenched in structural exploitation, weak regulation, and deliberate policy choices that restrict access to rights and protections. Taken together, these lenses raise two guiding questions: How do syndemic conditions interact with structural labor exploitation to affect worker health, and what health barriers do Thai agricultural migrant workers face in Israel? To address these questions, this study pursues three objectives: (1) to examine the health barriers experienced by Thai agricultural migrant workers in Israel; (2) to analyze how syndemic vulnerabilities intersect with structural labor exploitation to deepen inequalities; and (3) to identify policy interventions that can mitigate these disparities and promote more equitable health outcomes.

## 2. Methodology

This article results from collaboration between academic researchers and two civil society organizations: Kav LaOved (Workers’ Hotline) [55], which protects workers’ rights, and Physicians for Human Rights–Israel, which advocates for health equity for marginalized populations. The academic researchers were actively involved in designing the questionnaire, providing feedback, and monitoring the data collection process, ensuring methodological rigor and alignment with the research objectives.

The empirical basis of the study draws primarily on three surveys conducted between 2017 and 2020 by these organizations, complemented by qualitative data from worker inquiries and affidavits collected by Kav LaOved.

The surveys used a descriptive questionnaire method. They were translated into Thai by Kav LaOved and distributed via a Kav LaOved Facebook group widely used by Thai migrants in Israel (about 15,000 followers). This enabled broad outreach but limited the sample to active social media users. Still, the method is standard and reliable in social sciences for identifying phenomena. Participation was anonymous and approved by an Ethics Committee. Given the time frame, it is unlikely that the same individuals responded more than once. Respondents were also invited to contact Kav LaOved for assistance.

In addition to survey data, the study analyzed affidavits submitted by Thai agricultural workers that document their living conditions. Each affidavit was coded thematically to identify recurring patterns related to labor rights violations, employer dependence, and health-system barriers. The themes derived from these affidavits were triangulated with survey results to enrich interpretation and validate quantitative findings. Illustrative excerpts were included in the Results Section to present workers’ voices and contextualize statistical trends. To ensure the validity and contextual accuracy of the findings, descriptive survey results were cross-checked with qualitative evidence drawn from workers’ affidavits collected by the NGOs and from the researcher’s qualitative fieldwork.

A potential self-selection bias may exist, as respondents were likely those especially interested in the topic. To mitigate this limitation, the surveys were distributed through multiple channels, including social media and direct outreach by community organizations, in order to reach workers with varied levels of engagement and awareness. Although participants may represent those more informed about their rights, the patterns identified, such as recurring language barriers, unsafe housing, and limited access to healthcare were consistent across independent data sources, providing a reliable picture of systemic barriers rather than individual perceptions.

While the study’s design allows for a broad descriptive mapping of migrant workers’ health barriers, several limitations must be acknowledged. The cross-sectional nature of the data precludes causal inference, and some responses may reflect recall or interpretation biases due to translation and cultural differences. Finally, because the study focused mainly on Thai agricultural workers, its findings may not be generalizable to all migrant populations or sectors. Despite these constraints, triangulation between survey data and affidavit narratives enhances validity and provides a robust foundation for understanding how structural, legal, and health-system factors intersect to shape migrant workers’ well-being.

Table 1 depicts the different characteristics of each survey presented in this research.

The surveys were designed to capture key aspects of migrant workers’ health as outlined above. Each questionnaire included approximately 30 questions. The topics were selected by the NGOs according to field demands at the time. The vast majority of respondents were men, reflecting the overall gender composition of the sector-approximately 97% of agricultural migrant workers are male. Participants were between 20 and 40 years old, and most had been residing in Israel for more than two years.

The study followed a grounded theory orientation, in which theoretical insights emerged inductively from the empirical data. The surveys were conducted prior to the articulation of the theoretical framework. Subsequent analysis identified recurring patterns that informed the integration of the syndemic and labor approaches, rather than these frameworks guiding the initial survey design.

## 3. Results

The analysis identified three interrelated themes that illustrate how structural conditions shape the health of Thai agricultural workers in Israel: (1) barriers to healthcare accessibility, including linguistic and institutional obstacles; (2) poor living and working conditions that exacerbate physical risks; and (3) mental health challenges linked to isolation, overwork, and insecurity.

### 3.1. Structural and Linguistic Barriers to Healthcare Accessibility

Workers described serious difficulties with accessing medical services, even when formally insured. As one worker testified, “I was never given a health fund membership card… A doctor was never brought in, and it was never explained to us that this was even possible.” Survey data confirms that 52% of injured workers did not seek medical treatment, mainly due to employer restrictions or lack of insurance awareness. Language barriers further limited access. Among those who sought care, 44% could not understand the physician because no translation was provided, and 43% reported difficulties exercising their right to medical services. As a result, nearly all respondents (98%) preferred using medicine brought from Thailand or sent by relatives. Some even produced improvised remedies from local materials.

These practices reveal how structural exclusion and linguistic isolation intersect effective treatment. Limited translation, lack of insurance documentation, and dependence on employers leave workers without meaningful access to Israel’s healthcare system.

### 3.2. Living and Working Conditions

The surveys depict severe shortcomings in the workers’ living environment. Many are housed in makeshift structures that lack basic facilities, adequate space, or proper ventilation. About one-fifth reported having no windows, and more than half lacked sufficient heating, cooling, or bedding. Overcrowding was widespread, with an average of ten workers sharing each unit; in some cases, up to fifty people were housed in a single structure. Access to clean drinking water was limited, and improvised gas and plumbing connections posed additional safety hazards. These conditions, far below legal housing standards, exposed workers to constant discomfort and heightened health risks.

Agricultural work is physically demanding and often performed under extreme weather conditions. Many participants reported excessive hours and lack of rest days. As one worker explained, “The work is very hard… I was employed 11 h a day… without a single day of rest.” According to the surveys, 18% of Thai migrant workers reported working more than 12 h daily, and about 60 percent felt constantly tired. Prolonged fatigue increased the risk of workplace accidents: 37% of respondents were injured at work. Exposure to pesticides was particularly hazardous—39% reported skin problems, 21% respiratory difficulties, and 20% eye irritation or stomach pain. Only one third received protective equipment from their employers, while 24% worked without any and 40% purchased their own. Together, these findings reveal how overwork, chronic fatigue, and unsafe practices interact to endanger workers’ health.

### 3.3. Mental Health

Migrant agricultural workers are exposed to many stressors stemming from the range of factors described above—including inadequate housing conditions and the heavy workload under harsh and life-threatening conditions, together with various aspects of the migration experience itself, such as uprootedness and distance from home and community. Accordingly, in the Health Survey, nearly half of the workers (46%) reported psychological distress.

The psychological hardship and the inability to receive support is illustrated in the testimony of K.T., a Thai migrant worker: “In the past month, I experienced a severe mental crisis. A serious incident occurred in my family in Thailand, along with an economic crisis, and other issues that are hard for me to talk about. (…) I told my employer that I had a problem in Thailand. I tried to speak with him in Hebrew, which is difficult. I did my best to explain, but the employer only replied, ‘I have no money to give you.’” (Affidavit by K.T. for the purpose of regulating the worker’s legal status (February 2019)).

## 4. Discussion

This study demonstrates how the health of Thai migrant agricultural workers in Israel is shaped by the intersection of syndemic and structural forces. Rather than isolated occupational or psychological problems, the findings reveal an integrated system in which legal exclusions, unsafe labor conditions, and social marginalization interact to amplify vulnerability. As we saw in the Findings section, we find the syndemic coexistence of work related diseases such as skin problems or respiratory problems, work injuries, and psychological distress. From a syndemic perspective, these intersecting exposures—physical, environmental, and psychosocial—mutually reinforce one another, producing cumulative health harm: for example, the coexistence between exposure to pesticides, fatigue and poor living conditions increase the risk for respiratory diseases, which can be worsened by obstacles in access to healthcare services. The labor framework clarifies how these health patterns are embedded in institutional design, where dependence on employers, weak enforcement, and privatized health insurance constitute structural mechanisms of exploitation.

Interpreting the results through these two lenses allows a deeper understanding of how state policy and market arrangements co-produce health inequities. Workers’ exhaustion, injuries, and mental distress are not random outcomes but predictable results of a system that externalizes health costs while maximizing labor extraction. Therefore, health vulnerabilities among migrant workers cannot be mitigated solely through clinical or behavioral interventions but require structural reform in labor and health governance.

From a syndemic perspective, the data points to an ecosystem in which occupational injuries, psychosocial trauma, language barriers, and institutional neglect co-occur and interact within a hostile social and legal environment. These intersecting vulnerabilities do not merely accumulate but amplify one another, producing a cascade of negative health outcomes that extend beyond the workplace and into the broader social fabric of migrant workers’ lives. As Singer et al. (2021) argue [56], syndemics are exacerbated by environments of exploitation and inequality—precisely the conditions facing MLWs in Israel’s agricultural sector. In this case, the syndemic framework underscores how structural determinants, such as inadequate housing or exclusion from public health services, become catalysts that intensify the physical and mental health toll of pesticide exposure, overwork, and social isolation.

The labor framework clarifies how Israel’s legal and institutional settings translate structural dependence into health vulnerability. By delegating responsibility for health insurance to employers and private companies, the state creates a system in which access to care is conditional and uneven. Workers’ reliance on employers for medical documentation or translation deters them from seeking treatment after injuries and reinforces chronic under-reporting of occupational accidents. Limited enforcement and fragmented oversight further normalize unsafe practices such as extended working hours, lack of rest days, and inadequate housing that directly contribute to fatigue, pesticide exposure, and psychological stress. In this way, legal arrangements designed to regulate migration and employment become central determinants of health inequality.

Seen together, the syndemic and labor approaches bring into focus a broader paradox: although migrant workers are essential to maintaining Israel’s agricultural economy, they remain politically invisible and institutionally marginalized. This contradiction raises further questions for policy and research. How can occupational health monitoring systems be designed to capture the cumulative effects of multiple, interacting risks rather than treating them as isolated problems? What role could transnational agreements between Israel and sending countries, such as Thailand, play in creating binding standards for health and safety protections? Moreover, how might collective worker agency through informal networks, social media, or cross-border advocacy serve as a counterweight to the structural coercion embedded in the current system? These questions point toward future directions that move beyond documenting vulnerabilities to exploring pathways of resistance, resilience, and systemic reform.

The structural dynamics discussed above are not unique to Israel but reflect a broader global pattern in which migration policy and labor regulation jointly determine workers’ health outcomes. For example, comparative evidence from Canada and the United States reveals similar patterns of structural vulnerability. In Canada, migrant farmworkers often live in overcrowded housing and face barriers to healthcare that mirror those experienced by Thai workers in Israel [12,14]. In the United States, research among Mexican and Central American farmworkers shows that legal insecurity and exclusion from insurance systems intensify occupational injuries and psychological distress [44,45]. These parallels indicate that Israel’s case is not an exception but part of a global regime of temporary labor migration in which systemic exploitation and health inequities are embedded within policy design.

Thus, the Israeli case contributes to a wider comparative discussion on the intersection of syndemics and labor exploitation. It underscores the need for global policy innovations, including cross-border regulation, rights-based health inclusion, and the recognition of migrant worker agency, that address not only national labor markets but also the transnational forces shaping them. By situating the health experiences of Thai agricultural workers in Israel within both local structural dynamics and comparative global patterns, this study demonstrates that combining the syndemics and labor frameworks offers a powerful analytical lens: it reveals how health inequalities are not merely clinical or individual outcomes but systemic products of intersecting vulnerabilities and structural exploitation, a reality that resonates far beyond Israel’s borders.

It is important to note that the data analyzed in this article was collected prior to the 7 October massacre and the war that followed it, which profoundly affected the Thai migrant worker community in Israel. On 7 October, 39 Thai workers were killed and 31 abducted to Gaza. Of those abducted, 26 returned alive and five were killed in captivity. Seven additional Thai workers lost their lives during the ongoing war [51]. Beyond academia, this study informs Israeli debates on labor migration, health policy, and social responsibility. By documenting Thai farmworkers’ experiences, it challenges their political invisibility and frames them as integral to Israel’s economy and moral fabric. The findings reveal systemic failures and underscore Israel’s ethical duty to protect migrant workers’ health, dignity, and security.

This research contributes to the Israeli context by bridging scholarship, policy, and civil-society advocacy. It provides evidence-based insights that can guide policymakers in revisiting the exclusion of migrant workers from the National Health Insurance Law, ensuring occupational health monitoring, and strengthening enforcement mechanisms. Ultimately, the study’s main contribution lies in demonstrating how the integration of the syndemics and labor frameworks reveal health inequalities as systemic outcomes of legal and institutional design, offering a model for analyzing and addressing migrant health vulnerabilities in other contexts.

## 5. Limitations

This study has several limitations that should be acknowledged. First, the data was collected through voluntary online surveys and affidavits, which may reflect a self-selection bias: participants who chose to respond could be those more aware of their rights or more motivated to report poor conditions. Second, because the surveys were cross-sectional and descriptive, they cannot establish causal relationships between specific policy mechanisms and health outcomes. Third, language barriers and cultural factors may have influenced workers’ interpretation of survey questions, despite careful translation into Thai. Fourth, the data was gathered prior to the 7 October war, and subsequent changes in Israel’s labor and security context may have affected migrant workers’ conditions in ways not captured here. Finally, as the study focused primarily on Thai agricultural workers, its findings may not be generalizable to other migrant populations or sectors. Nevertheless, these limitations do not detract from the study’s contribution to understanding how structural, legal, and health-system factors interact to shape migrant workers’ well-being.

## 6. Conclusions and Future Directions

This study identifies how Thai agricultural migrant workers in Israel experience overlapping health vulnerabilities arising from exclusionary labor and health-insurance systems. By integrating the syndemics and labor frameworks, it shows that workers’ physical and mental health risks are not isolated problems but products of institutional design and policy neglect. Future reforms should therefore focus on systemic accountability and inclusive health coverage to ensure safer and more equitable conditions for all migrant laborers.

Based on these findings, we propose a set of integrated recommendations addressing regulatory, health, and social dimensions of migrant worker protection.

## 7. Policy and Practice Recommendations

Regulatory measures: Strengthen labor inspection mechanisms and apply meaningful sanctions for noncompliance as well as incentives to improve the current situation. Ensure employer accountability through transparent reporting and coordinated oversight across ministries responsible for labor, health, and welfare.

Health-system reforms: Incorporate migrant workers into the National Health Insurance Law or mandate equivalent coverage through public schemes. Establish occupational health surveillance programs that include seasonal and pesticide-related monitoring, and guarantee access to multilingual medical services.

Social and community support: Provide psychosocial counseling and cultural mediation services to address isolation and mental distress. Support worker-led networks and collaborations between NGOs and municipalities to enhance awareness of rights and improve living conditions and social determinants of health.

In doing so, the study advances understanding of how legal and labor structures shape health inequalities, offering a conceptual and policy model for improving protection of migrant workers globally. Both collaborating organizations, as well as the academic researchers involved, have previously acted, and continue to act, to implement these recommendations in practice. Translating research findings into evidence-based policy proposals is an integral part of their ongoing advocacy and scholarly work, which includes preparing shadow reports submitted to international bodies and other monitoring mechanisms.

## Figures and Tables

**Table 1:** List of surveys.

Survey Name	Year	Main Topics	Number of Respondents
Pesticides Survey	2017	Demographic aspects, working with pesticides, work accidents, enforcement	190
Health Survey	2019	Medical condition before and after arrival in Israel, knowledge of health insurance rights, working and living conditions, relationship between conditions and health	130
Work and Living Conditions Survey	2020	Working and living conditions	178

## Data Availability

Available upon request.
